# Supplementary material for: Frontline Screening for SARS-CoV-2 Infection at Emergency Department Admission by Third Generation Rapid Antigen Test: Can We Spare RT-qPCR?
Source: Viruses. 2021 May 1;13(5):818. doi: 10.3390/v13050818 (PMC8147338; doi:10.3390/v13050818)
Supplement: Supplementary file 1 [file viruses-13-00818-s001.zip › viruses-1142372-supplementary.pdf]

Supplementary Figure 1. Selection criteria for the 960 swab samples included in the study and concordance between RT-qPCR and LumiraDx.

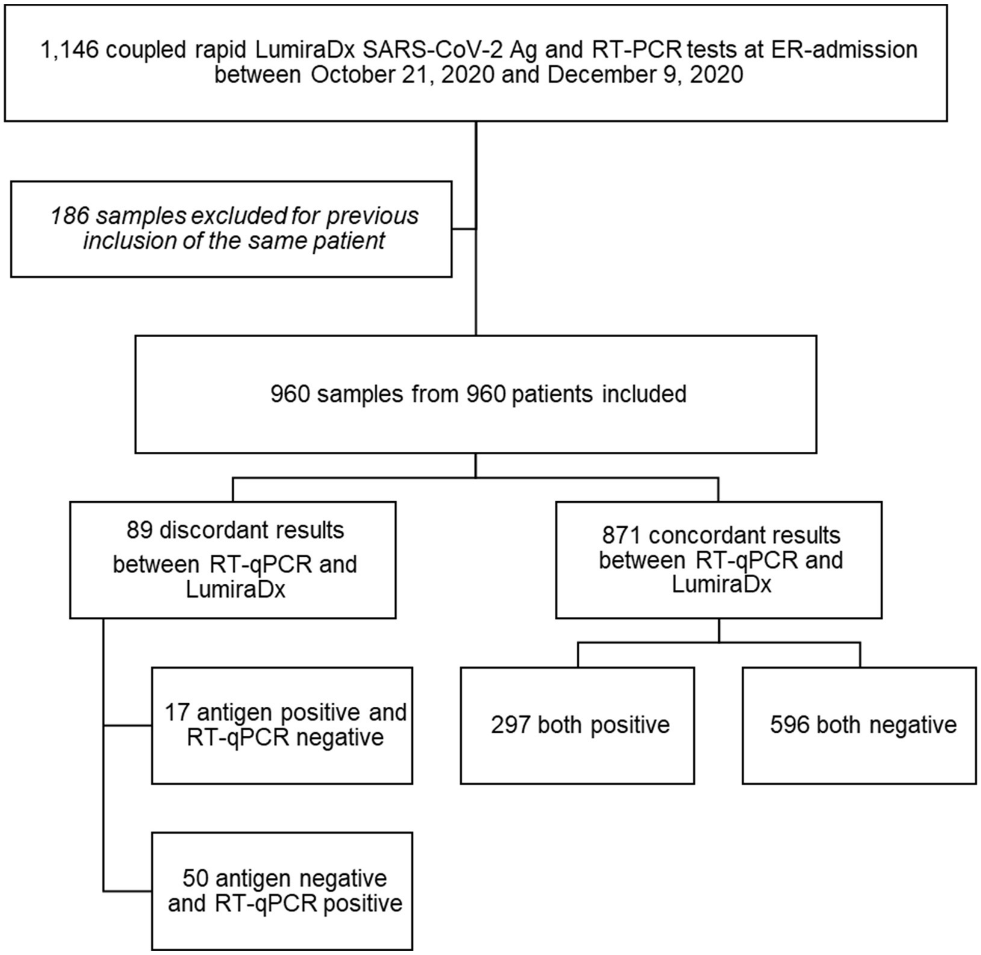

Supplementary Table 1. Factors associated with the presence of SARS-CoV-2 subgenomic RNAs

	**Presence of SARS-CoV-2 subgenomic E and N mRNA**			
	**Univariate analysis**		**Multivariate analysis**	
	**Odds ratio (95% CI)[a]**	**P-value**	**Odds ratio (95% CI)[a]**	**P-value**
**Delay between symptoms onset and SARS-CoV-2 diagnosis[b]**	0.25 (0.05-1.25)	0.092	2.74 (0.20-37.11)	0.449
**Severity of COVID-19 presentation, n (%)[c]**				
Mild (N=14)	Ref.	-	Ref.	-
Moderate (N=15)	0.51 (0.11-2.36)	0.392	1.01 (0.11-8.87)	0.991
Severe (N=29)	0.90 (0.17-4.70)	0.901	0.70 (0.05-9.48)	0.795
Critical (N=9)	0.20 (0.01-2.87)	0.237	0.11 (0.00-7.37)	0.308
**SARS-CoV-2 genomic RNA (copies/mL), median (IQR)[d]**	1.0 (1.0-1.0)	0.750	1.00 (1.00-1.00)	0.02

Odds ratio (95% CI) for subgenomic E or N mRNA production by univariate and multivariate logistic regression analysis are shown. [a] Related to the risk of being positive for E and/or N subgenomic mRNAs. [b] Self-reported and per day increase. [c] Classified according to WHO grading(18). [d] Per 1 Log_{10} increase. CI= Confidence interval; COVID-19= Coronavirus Disease 2019; IQR= interquartile range; N = number; SARS-CoV-2= Severe Acute Respiratory Syndrome Coronavirus 2.
